# Isolation of uracil auxotroph mutants of coral symbiont alga for symbiosis studies

**DOI:** 10.1038/s41598-018-21499-3

**Published:** 2018-02-19

**Authors:** Yuu Ishii, Shinichiro Maruyama, Konomi Fujimura-Kamada, Natsumaro Kutsuna, Shunichi Takahashi, Masakado Kawata, Jun Minagawa

**Affiliations:** 1 Department of Environmental Life Sciences, Graduate School of Life Sciences, Tohoku University, Sendai, Miyagi Japan; 2 Division of Environmental Photobiology, National Institute for Basic Biology, Okazaki, Aichi Japan; 3 Department of Integrated Biosciences, Graduate School of Frontier Sciences, University of Tokyo, Kashiwanoha, Chiba, Japan; 4 LPixel Inc., Bunkyo, Tokyo Japan; 5 Department of Basic Biology, School of Life Science, SOKENDAI (The Graduate University for Advanced Studies), Okazaki, Aichi Japan

## Abstract

Coral reef ecosystems rely on stable symbiotic relationship between the dinoflagellate *Symbiodinium* spp. and host cnidarian animals. The collapse of such symbiosis could cause coral ‘bleaching’ and subsequent host death. Despite huge interest on *Symbiodinium*, lack of mutant strains and readily available genetic tools have hampered molecular research. A major issue was the tolerance to marker antibiotics. Here, we isolated *Symbiodinium* mutants requiring uracil for growth, and hence, useful in transformation screening. We cultured *Symbiodinium* spp. cells in the presence of 5-fluoroorotic acid (5FOA), which inhibits the growth of cells expressing *URA3* encoding orotidine-5′-monophosphate decarboxylase, and isolated cells that require uracil for growth. Sequence analyses and genetic complementation tests using yeast demonstrated that one of the mutant cell lines had a point mutation in *URA3*, resulting in a splicing error at an unusual exon–intron junction, and consequently, loss of enzyme activity. This mutant could maintain a symbiotic relationship with the model sea anemone *Exaiptasia pallida* only in sea water containing uracil. Results show that the *URA3* mutant will be a useful tool for screening *Symbiodinium* transformants, both *ex* and *in hospite*, as survival in the absence of uracil is possible only upon successful introduction of *URA3*.

## Introduction

The dinoflagellate *Symbiodinium* spp. are known to sustain a stable symbiotic relationship with cnidarian animals (e.g. coral, sea anemone, jellyfish) by endosymbiosis in the gastroderm (endoderm) cells of host cnidarian animals^1^. Ecologically, *Symbiodinium* is a key primary producer for sustaining the coral reef ecosystems in the oligotrophic tropical and subtropical ocean, and much of the photosynthetically fixed carbon by symbionts is provided to the host coral^2^. Collapse of the coral-algal symbiosis, which is known as ‘bleaching,’ often leads to death of the host corals, causing destructive damage on the coral reef ecology^2,3^.

From a taxonomical perspective, although *Symbiodinium* spp. can be classified into a number of ‘clades’ by means of molecular phylogeny^4^, all these clades lack conspicuous morphological traits applicable for species-level classification. Recently, advances in sequencing technology have revealed the diversity of *Symbiodinium* across a range of coral reefs and other marine environments. Previous studies suggested that the specificity of the *Symbiodinium-*cnidarian symbioses was dependent on the size of the algal symbiont^5^, and that the symbiont specificity of corals increased (i.e. fewer *Symbiodinium* types can be associated with corals) as the host coral grew^6^. On the other hand, no substantial change on the symbiont specificity was observed in the model sea anemone *Exaiptasia pallida* (formerly *Aiptasia* sp.)^7^.

In spite of the accumulation of genomic and multi-omics information on cnidarian-algal symbiosis^8^, genetic tools for characterizing functions of genes that are involved in such symbiosis are still very limited and not readily available. Two independent studies on gene delivery into the *Symbiodinium* cells have been published. The first report by ten Lohuis and Miller discusses successful delivery of external DNA molecules using silicon carbide whiskers^9^. Seventeen years later, Ortiz-Matamoros and colleagues reported transient expression of exogenous genes delivered into *S. microadriaticum* subsp*. microadriaticum* strain S. KB8, *Symbiodinium* sp. strain Mf11.5b.1, and the genome-sequenced strain *Symbiodinium kawagutii*^10^, using polyethylene-glycol with glass beads^11^ or the terrestrial bacterium *Agrobacterium tumefaciens*, which has been widely used for transformation of land plants^12^. However, further elaboration of methods for gene delivery into *Symbiodinium* cells is clearly needed: No follow-up studies have been published using the methods developed by ten Lohuis and Miller^9^ and, although it was shown that transient gene introduction methods used for land plants could also be applicable to *Symbiodinium*, no stable transformant lines have been reported^12^.

Towards developing a genetic tool for the coral symbiotic dinoflagellate *Symbiodinium* sp., we conducted antibiotic screening experiments and isolated a nutrient (uracil)-requiring *Symbiodinium* mutant. We show that the cell growth could be switched on and off by replacing the media, and that the growth switching was inducible *in* and *ex hospite*.

## Results

### Screening and phenotyping 5FOA-resistant and nutrient (uracil) -deficient mutants

**Figure 1:**
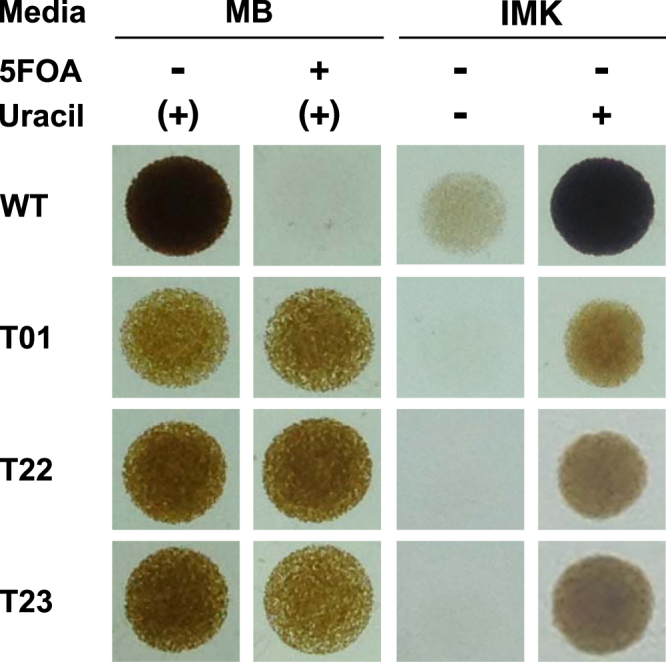
Phenotypes of *Symbiodinium* wild type and mutant cells. Wild type (WT) and mutant (T01, T22 and T23) cells were spotted and grown on agar plates with complete medium (MB), which originally contains uracil, with or without 5FOA, and the minimal medium (IMK) with or without uracil.

To select antibiotics and inhibitors suitable for genetic screening experiments, and that could regulate *Symbiodinium* cell growth by their presence or absence, we tested the effects of widely-used antibiotics including kanamycin, neomycin, streptomycin, zeocin, paromomycin and nourseothricin, on cell growth. None of the antibiotics tested had a substantial effect on the algal growth. However, a cell growth inhibitor 5-fluoroorotic acid (5FOA) successfully suppressed the algal growth (Fig. 1, WT). 5FOA is a fluorinated derivative of uracil precursor orotic acid and inhibits the growth of cells expressing *URA3* gene, which encodes orotidine-5′-monophosphate (OMP) decarboxylase, through the synthesis of the toxic 5-fluorouracil causing cell death (Supplementary Fig. S1).

This inhibitor has also been commonly used in yeast, red alga, and other organisms to isolate 5FOA insensitive, nutrient (uracil)-requiring mutants, i.e. *ura3* mutant^13^. In addition, only one copy of *URA3* gene in each of the sequenced genomes of *S. minutum*^14^ and *S. microadriaticum*^15^ was found, suggesting that *URA3* was more promising as a marker gene than multi-gene family members (Supplementary Fig. S2A). Thus we used 5FOA for further screening because this inhibitor was useful to establish cell lines whose growth could be regulated by the presence and absence of uracil. For isolating 5FOA-resistant mutants from *Symbiodinium*, we used the strain SSB01 (clade B phylotype), which is an axenic uni-algal strain closely related to *S. minutum* Mf1.05b (clade B), the strain whose genome has been sequenced^14,16^. We grew SSB01 cells in the nutrient-replete, uracil-containing Marine Broth (MB) medium containing 200 µg/ml 5FOA over eight weeks and successfully isolated four candidate mutant lines. We named these mutant lines T01, T22, T23 and T29, and confirmed that the three of them (T01, T22 and T23) showed stable and reproducible growth suppression in the uracil-limited sea salt medium (IMK) plates and resistance to 5FOA (Fig. 1).

### Sequence analysis of mutant strains

**Figure 2:**
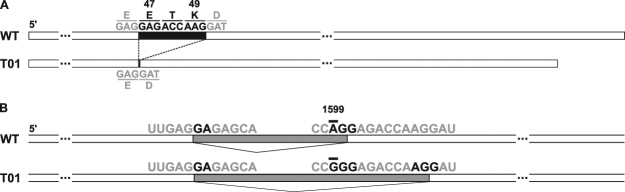
Sequences of *URA3* cDNA and splicing variation on mRNA between wild type and T01. (**A**) A deletion of 9 bp, corresponding to 3 amino acids (47-49th), found in the T01 *URA3* cDNA is indicated in a black closed box. (**B**) Grey boxes and bold letters represent introns and nucleotides relevant to intron-exon junction recognition, respectively. Letters with bars represent the nucleotide site where the mutation is mapped in the T01 genome.

To identify the mutation sites, we sequenced partial cDNA sequences (from 97th to 532th nucleotide in the 792-bp full length cDNA sequence) of the *URA3* gene of the mutant T01, T22, T23, T29 and wild type SSB01 strains. We found a 9-bp deletion corresponding to three amino acid sites (47-49th) in the T01 cDNA (Fig. 2A), while no mutations in the partial cDNA sequences were found in the strains T22, T23 and T29. Homology-based comparison with the crystal structure of the URA3 homolog from other species^17^ suggested that, although *Symbiodinium* and some other eukaryotes possess homologs only distantly related to well-studied URA3 proteins, this deletion site was proximal to a lysine residue, which is important for substrate recognition, and that the deletion likely affects the enzymatic activity resulting in resistance to 5FOA (Supplementary Fig. S2B).

**Figure 3:**
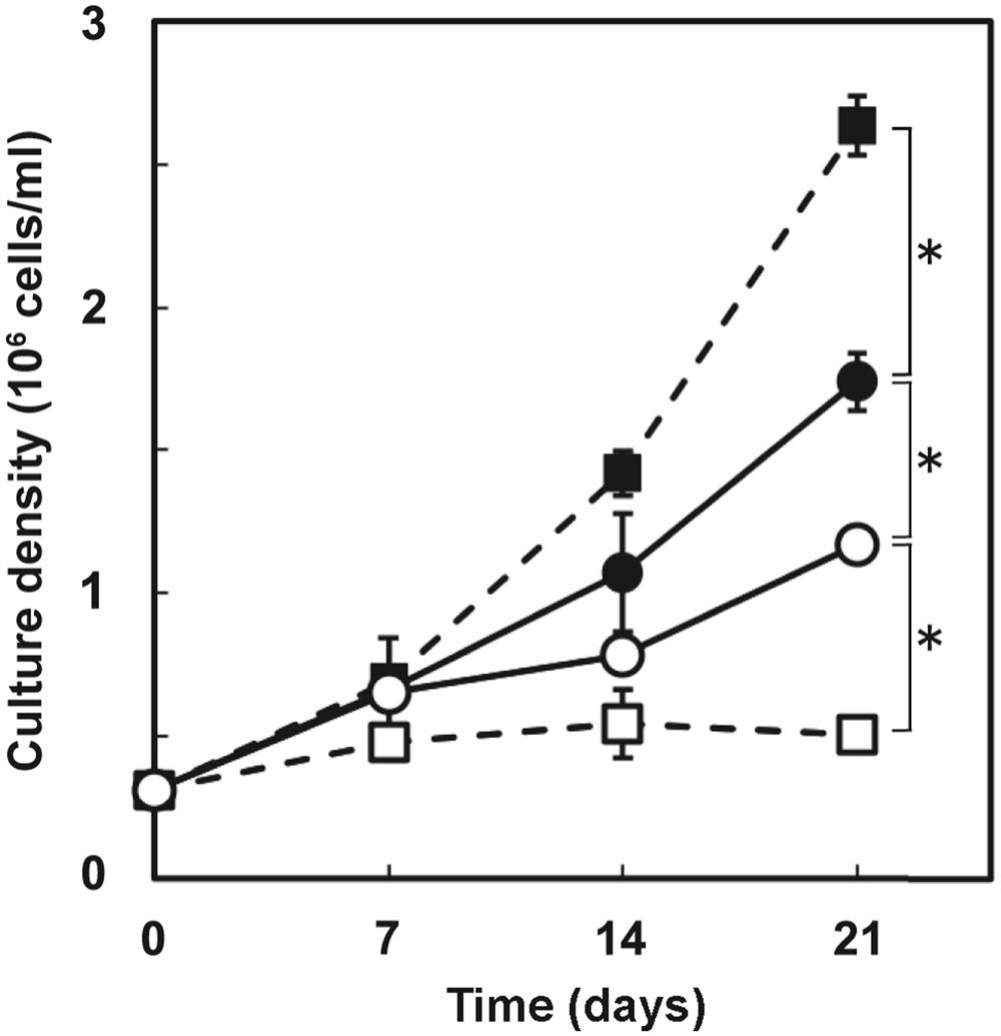
Effects of 5FOA on the cell growth rates of wild type and T01 in MB liquid cultures. WT (square) and T01 (circle) cells were grown in the absence (closed symbol) or presence (open symbol) of 5FOA (*adjusted p for interaction <0.05, n = 3, *t*-test).

To clarify the cause of the 9-bp deletion in the T01 *URA3* cDNA and search for potential mutation sites in the other mutants, we sequenced a 6-kb genomic DNA (gDNA) fragment containing the *URA3* gene, with reference to a scaffold sequence, scaffold311, and the gene models of the *S. minutum* genome database^14^. We identified a single nucleotide substitution in the intron region of an intron-exon junction in the T01 *URA3* genome (Supplementary Fig. S3, Supplementary data), while we found no mutations in the T22, T23 and T29 *URA3* genomic sequences in comparison to the wild type. By sequence comparison using our sequence data from the wild type SSB01 and T01, we found both the canonical intron-exon boundary sequences (‘GU-AG’) and non-canonical ones within the *URA3* gene, as previously shown in the *S. minutum* genome^14^. The single nucleotide substitution found in this study could alter a non-canonical ‘GA-AG(G)’ exon-intron junction into ‘GA-GG(G)’ (Fig. 2B), resulting in a splicing variation mutation. To further examine the phenotype-genotype correlations in SSB01 and T01, we cultured them in liquid media in the presence or absence of 5FOA. The growth of T01 was confirmed upon addition of 5FOA (Fig. 3), suggesting that the phenotype of T01 was the consequence of mutation(s) affecting uracil synthesis in the pyrimidine biosynthesis pathway (see Discussion and Supplementary Fig. S1).

### Yeast mutant complementation

**Figure 4:**
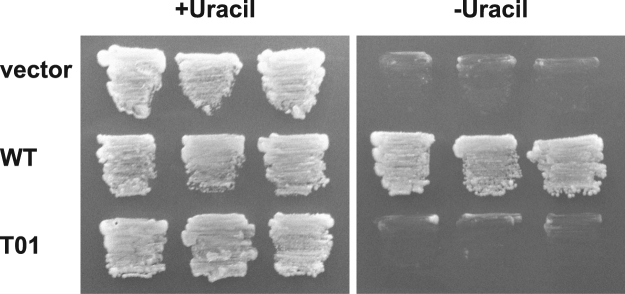
Functional complementation of *Symbiodinium URA3* genes in a yeast *ura3* mutant. The growth of the yeast *ura3* mutant cells transformed with an empty vector (vector), the wild type *URA3* cDNA (WT) and the mutant cDNA (T01) on plate containing (+Uracil) or lacking uracil (−Uracil).

Our sequence analyses showed that a single nucleotide substitution was the most likely reason for mis-splicing and the 9-bp deletion in the *URA3* gene at the cDNA level, resulting in 3-amino acid deletion at the protein level (Fig. 2). To confirm that the 3-amino acid deletion was responsible for the 5FOA-sensitive and uracil-requiring phenotypes, we conducted yeast complementation assays. For this assay, we cloned the wild type and the mutant (T01) cDNA sequences encoding *Symbiodinium* URA3 proteins with codon usage optimized for the budding yeast *Saccharomyces cerevisiae* into the vector p414-TEF, transformed a *ura3* mutant yeast cell line with them, and compared the growth on uracil replete and limited media (Fig. 4). While the wild type gene rescued the growth of the yeast cells on the uracil-limited medium, the vector control and the T01 gene did not recover the growth of the *ura3* yeast cells.

### Symbiosis experiments using the model sea anemone *Exaiptasia pallida*

**Figure 5:**
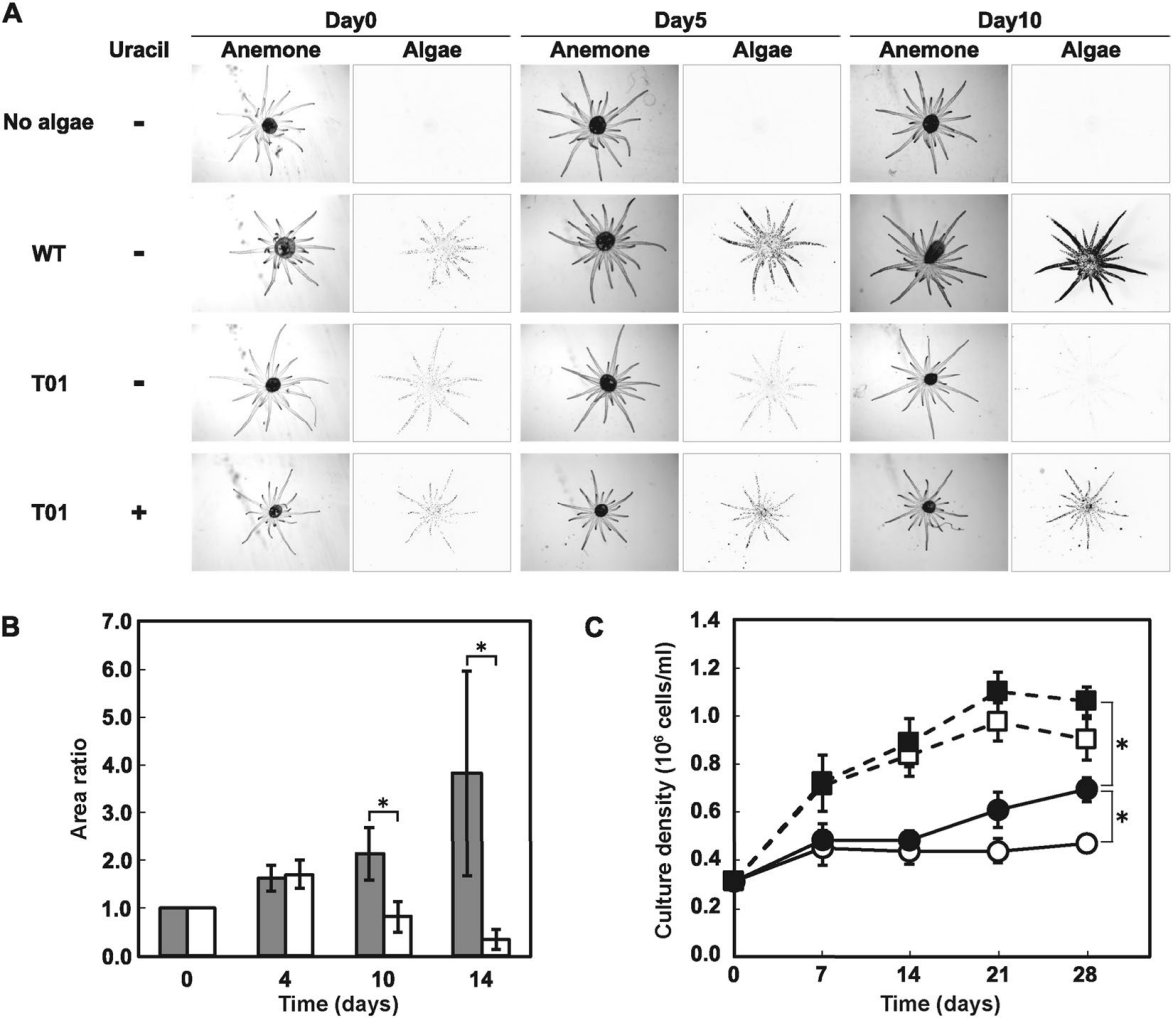
Symbiosis experiments using wild type and T01 cells. (**A**) Stereomicrographs using bright field (Anemone) or a green excitation filter set (Algae: chlorophyll autofluorescence of *Symbiodinium*) photographed with time intervals shown above. Representative images of individuals are shown for each treatment. (**B**) Effect of uracil on the symbiotic state of T01. *Symbiodinium* area ratios were quantified by comparing signal regions of chlorophyll and normalized to the value of day 0 in the presence (closed bar) or absence (open bar) of uracil (*p < 0.05, n = 3, *t*-test). (**C**) Effects of uracil on the cell growth rate of WT and T01 in ASW liquid culture. WT (square) and T01 (circle) cells were grown in the presence (closed symbol) or absence (open symbol) of uracil (*adjusted p for interaction <0.05, n = 3, *t*-test).

In order to examine whether the mutant strain T01 maintained the ability to symbiose with host cnidarian animals, we fed the model sea anemone *E. pallida* with T01 and wild type *Symbiodinium* cells. Then we calculated ‘area ratio,’ which is defined as the ratio of the area having chlorophyll autofluorescence signal of the intracellular algal cells in micrographs taken from the top of the sea anemone at regular time points in comparison to the one at the start of the quantification (day 0), which roughly represented the degree of symbiosis expansion in the host animal body. In the artificial seawater (ASW) medium supplemented with uracil, the area ratio in the *E. pallida* fed with T01 increased over incubation time and was stably sustained, indicating that T01 retained the ability to establish a symbiotic relationship with *E. pallida* in the uracil-replete condition, as the wild type strain did in the uracil-depleted condition (Fig. 5). However, when the uracil-containing ASW was replaced with the one lacking uracil, the number of T01 cells living inside the animal host gradually decreased over time, while T01 retained stable symbiosis when the medium was replaced with fresh uracil-containing ASW (Fig. 5A, B, Supplementary Fig. S4). The area ratio of T01 in the uracil-replete condition increased gradually compared to the wild type in uracil-lacking ASW (Supplementary Fig. S4), and the rate of increase in the area ratio seemed to reflect the growth ability of the free-living cells (Figs 3 and 5C) (see Discussion). These results clearly demonstrate that stable symbiotic relationship between *E. pallida* and T01 was dependent on the availability of uracil in the environment, which is critical for the growth of the algal symbiont (Fig. 5C).

## Discussion

Cnidarian-dinoflagellate symbiosis is one of the well-studied and unique model systems that can be used for examining cellular mechanisms of animal-plant symbiosis^18^, but has not been understood fully at the molecular level. The availability of *Symbiodinium* strains possessing conspicuous physiological and/or cellular properties enable easy tracking in symbiosis experiments, and could be an ideal genetic tool. As such no such strain has been available until now. In this study we identified a nutrient-requiring mutant strain of *Symbiodinium* harbouring spontaneous mutations in a gene encoding the uracil synthesis enzyme. We also demonstrated that this mutant can be employed for analysing cellular properties in symbiosis experiments.

It is worth noting that the nutrient-requiring mutants, similar to those screened in this study, serve as potentially useful tools for researchers to develop systems for genetic transformation of *Symbiodinium*. Although gene introduction methods for *Symbiodinium* have been reported^9,11,12^, several challenges including low reproducibility, difficulty in isolation, and recovery of actively-growing transformed cells prevent them from being routinely used. The T01 mutant line developed in this study will be a useful tool to examine whether a gene of interest affects the stability of symbiosis by transformation with a construct containing the target gene and wild type *URA3* either by fusion or as tandemly arranged genes to complement the uracil-requiring phenotype and not by mere transfection of exogenous DNA.

The mutant strains obtained in this study were viable on the medium containing both 5FOA and uracil, which strongly suggests their inability to synthesize uracil due to *URA3* gene mutation and/or suppressed gene expression (Fig. 1). Sequencing the cDNA confirmed the 9-bp deletion corresponding to 3-amino acids in the T01 mutant (Fig. 2A). By referencing the *Bacillus subtilis* URA3 protein structure^17^, the deletion region was predicted to be in the vicinity of a helix containing a lysine residue shown to be important for enzymatic activity (Supplementary Fig. S2B). This is consistent with the results of the yeast complementation tests (Fig. 4), indicating that the mutant *URA3* gene sufficiently explains the T01 phenotype and that the spontaneous *Symbiodinium URA3* mutant was successfully isolated through the 5-FOA resistance screens. The decreased cell growth rate of T01 compared to wild type strain even in the uracil-replete condition suggested that T01 might have other uncharacterized mutation(s) in the genes associated with growth rate regulation or that a slow growth individual was randomly selected (Fig. 3). Once the sexual reproduction cycle of *Symbiodinium* is fully characterized, further sophistication of algal genetic techniques, e.g. mating, backcrossing, as routinely done in the model green alga *Chlamydomonas reinhardtii*, will be useful to segregate mutations associated and not associated with the uracil-requiring phenotype and ‘purify’ the mutant strain^19^.

Sequence comparison of the genomic DNA of the *URA3* gene in T01 and wild type (SSB01) based on the genome database of the reference strain *S. minutum* Mf1.05b^14^ revealed a single nucleotide substitution in an intron (Supplementary Fig. S3). A previous genome study^14^ suggested that many *S. minutum* genes, including *URA3*, possess divergent atypical exon-intron boundary structures, and that the canonical splicing donor site (GU) and acceptor site (AG) sequences were not necessarily conserved. The intron junction sequence where we identified the nucleotide substitution did not follow the ‘GU-AG’ rule, but was found to be ‘GA-AG’ in the wild type strain. Interestingly, the mutation in T01 had substituted the acceptor ‘AG’ with ‘GG,’ resulting in defective mRNA splicing. The new acceptor was not the first ‘AG’ that was positioned downstream to the original acceptor but the second ‘AG’ (Fig. 2B). The original and second downstream ‘AG’ were followed by a ‘G,’ in contrast to the first downstream ‘AG’ followed by an ‘A,’ indicating that the splicing junction was probably recognized as ‘GA-AG-G’ including the first nucleotide of the downstream exon. To our knowledge, this is the first study using a splicing variant mutant of *Symbiodinium*, illustrating the unusual exon-intron boundary recognition functioning *in vivo*, as had been predicted in the genome analysis^14^.

Our results invoke further questions on the evolution and regulation of such uncanonical splicing mechanisms. First, the mechanisms of junction site recognition and determination remain unknown; e.g. the potentially recognizable accepter ‘AGG’ sequence was also located upstream to the original site but was not recognized for splicing^14^. Second, it is still unclear whether the nucleotide sequences are sufficient for determining the junctions or if other factors such as spliceosomal RNA and proteins are involved^20^. Recent advances in high throughput sequencing technology may be helpful in tackling these issues. Accumulating large amounts of transcriptomic and proteomic data from *Symbiodinium* culture strains and environmental samples can be useful in identifying natural variations in splicing junctions in conserved proteins. This will enable us to estimate how frequently acceptable protein sequence alterations resulting from splicing variations occur^21,22^. Biochemical analysis of dinoflagellate spliceosomes as well as genetic transformation system using the *Symbiodinium* mutant strains developed in this study will also be of great assistance in understanding the evolution of such complex and unusual splicing mechanisms in dinoflagellates.

Our co-culture experiments showed that T01 was able to maintain a stable symbiotic relationship with the model sea anemone *E. pallida* in uracil-containing ASW, as well as the wild type strain (Fig. 5), indicating that in T01 the cellular machinery involved in the symbiosis was not impaired. However, the symbiotic status between *E. pallida* and T01 became unstable when it was cultured in uracil-free medium (Fig. 5A and B), suggesting that the availability of uracil in the medium was a requisite for sustaining the stable symbiosis. Ten days after depletion of uracil, the symbiosed T01 cells were only sparsely distributed in the anemone body (Fig. 5A), which appeared to mimic the ‘bleaching’ status^22,23^. The unsuccessful symbiosis in the uracil-depleted condition suggested that the supply of uracil from the host to the symbiont was not enough, if any, to sustain the proliferation of the symbiont. Further, with the use of the mutant T01, it is now possible to experimentally ‘switch on and off’ the sea anemone-algal symbiosis by using media containing or lacking uracil, respectively. This has important implications on the relationship between symbiosis stability and the growth ability of the symbiont cell. In the free-living condition, T01 cell cultures showed the significantly increased cell growth depending on the availability of uracil 28 days after the onset of the medium change (Fig. 5C). Although it is difficult to directly compare the symbiotic and free-living conditions, the uracil-dependent cell proliferation in the free-living condition can explain to some extent how the symbiosis was established in the uracil-dependent manner. A plausible interpretation is that a certain level of cell proliferation is necessary for sustaining symbiosis. Considering that dividing *Symbiodinium* cells were preferentially expelled from cnidarian hosts^24^, even though a certain number of cells are expelled, their daughter cells can be re-symbiosed with the host after cell division resulting in the expansion of symbiosis if they outnumber the originally expelled cells. In the case of uracil-depleted T01 unable to proliferate without uracil, the number of cells re-entering the host endodermal cells decrease, thereby, leading to loss of algae inside the animal host (Fig. 5).

It should be noted that, although uracil was not supplied, or if any very limited, from the host to the symbiont in the model sea anemone *E. pallida*, other cnidarian hosts including corals may have different metabolic properties and are to be examined in future studies. This suggests that, although cautions are needed in interpreting results under less controlled experimental conditions, e.g. in the field or aquarium tank, the mutant strains developed in this study would be useful for studying metabolic interactions between hosts and symbionts, and also for screening host cnidarian species which supply uracil and possibly other basic metabolites to symbionts.

Cnidarian-algal endosymbiosis has been an important study model in ecology, genomics and cell biology due to its huge impact on marine ecosystems, especially in tropical and subtropical areas^1^. Previous studies have shown that elevated sea water temperature could lead to the collapse of symbiosis and coral ‘bleaching’^25–27^. Thus, understanding the mechanisms of maintaining stability of symbiosis is key to predicting possible effects of environmental changes on marine ecosystems. To our knowledge, the first mutant strain of *Symbiodinium* established in this study emphasizes the importance of cell proliferation in sustaining the symbiosis *in vivo*, and can be used to investigate the molecular mechanisms of the symbiosis in future studies. This will be a powerful tool for *Symbiodinium* genetics research, and for advancing ‘symbiotic genetics’, through which it is possible to examine what kinds of genes are relevant for establishing stable symbiotic relationships with cnidarian hosts and for using genetically engineered symbiotic algae.

## Materials and Methods

### Culturing and screening methods

*Symbiodinium* strain SSB01, a generous gift from Profs. John R. Pringle and Arthur R. Grossman, was an axenic uni-algal strain closely related to the genome-sequenced strain *S. minutum* Mf1.05b (clade B)^14,16^. SSB01 was maintained at 25 °C in Marine Broth (MB) medium containing 33.5 g L^−1^ of MB (Difco Laboratories, New Jersey, USA), 250 mg L^−1^ of Daigo’s IMK Medium (Nihon Pharmaceutical, Japan), and PSN (Gibco, Thermo Fisher Scientific, Massachusetts, USA) where the final concentrations of penicillin, streptomycin and neomycin were 0.01, 0.01, 0.02 mg ml^−1^, respectively. Light was provided at an irradiance of approximately 100 µmol photons m^−2^ s^−1^ in a 12 h light: 12 h dark cycle. IMK medium, which contained 33.5 g L^−1^ of Sea salt (Sigma-Aldrich, Merck Millipore, Germany), and 250 mg L^−1^ of Daigo’s IMK Medium, was also used in some experiments.

To obtain spontaneous 5FOA-resistant mutants, approximately 1 to 5 × 10^7^ cells of SSB01 were concentrated by centrifugation and spread on each MB plate (90 mm dish) containing 1% Agar (Wako Pure Chemical Industries, Japan) and 200 µg L^−1^ of 5FOA. After all, four independent clones were isolated from eight screening plates after 2 months of incubation under a 12 h light: 12 h dark cycle, with lighting provided at an irradiance of 5-20 µmol photons m^−2^ s^−1^, at 25 °C. After subsequent culturing, we designated these clones showing stable growth in the liquid media as T01 (Tohoku University strain 01), T22, T23 and T29.

To examine the 5FOA-resistant and uracil-requiring phenotypes, we grew SSB01 and T01, T22, T23 on MB and IMK plates either containing or lacking 5FOA. The cells were spotted at the concentration of 2.5 × 10^2^ cells/10 µl on MB plate followed by 4-week incubation, and 8.75 × 10^2^ cells/10 µl on IMK plate followed by 10-week incubation. To compare the growth of SSB01 and T01, we cultured these strains in liquid media. For liquid cultures using MB and artificial seawater (ASW) using Instant Ocean sea salt (Aquarium systems, France), the cells were inoculated in 20 ml of media (3.125 × 10^5^ cells ml^−1^) in T25 flasks. The cell growth was measured by counting the number of cells using TC20 Automated Cell Counter (Bio-Rad Laboratories, California, USA). Student’s two-tailed t-tests were performed to test whether the growth rates significantly differed between the strains and treatments.

For antibiotics resistance tests, we grew *Symbiodinium* strains CCMP 830 (clade B), 2429 (clade A), 2434 (clade B) and 2455 (clade F) on IMK plate, where the concentration of the sea salt was reduced to half to increase the sensitivity to antibiotics, containing 1% agar and 0.1 mg ml^−1^ ampicillin plus either of the following antibiotics; 0.2 mg ml^−1^ kanamycin, 0.4 mg ml^−1^ neomycin, 1.0 mg ml^−1^ streptomycin, 0.2 mg ml^−1^ paromomycin, 0.2 mg ml^−1^ nourseothricin (Sigma-Aldrich, Merck Millipore, Germany) and 0.2 mg ml^−1^ zeocin (Thermo Fisher Scientific, Massachusetts, USA).

### RNA extraction and determining the *URA3* transcript sequence

RNA was extracted from SSB01 and T01, T22, T23, T29. Cells were collected by centrifugation at 13,000 × g for 1 min at 25 °C. Culture medium was discarded, and the cell pellet was re-suspended in 500 µl of TRIzol (Thermo Fisher Scientific, Massachusetts, USA) and approximately 20 µl of Glass beads (Sigma-Aldrich, Merck Millipore, Germany) were added and mixed well by vortexing, followed by addition of an equal volume of chloroform and vortexing. After centrifugation and separation, the aqueous phase was used to purify total RNA by RNeasy Mini kit (Qiagen GmBH, Germany) following the manufacturer’s protocol. For synthesizing cDNA, High-Capacity cDNA Reverse Transcription Kit (Thermo Fisher Scientific, Massachusetts, USA) was used with random primers according to the manufacturer’s protocol. The URA3 cDNA was amplified by PCR using semi-nested PCR with three primers (1^st^, Ura3c_F1 and Ura3c_R1; 2^nd^, Ura3c_F1 and Ura3c_R2) (Table S1) using Tks Gflex DNA polymerase (Takara Bio, Japan). The product of the second PCR was tailed with adenine using *Taq* polymerase (Takara Bio, Japan) and cloned into pGEM-T Easy Vector Systems (Promega, Madison, USA). The plasmids were sequenced using ABI 3130 DNA sequencer (Applied Biosystems, California, USA) and using three primers (T7, SP6m and Ura3_R2).

### DNA extraction and determining *URA3* gene sequence

To determine SSB01 and T01 *URA3* gene sequences, we used the genome sequence of *S. minutum* strain Mf1.05b for reference^14^. As the gene model scaffold311.1|size231230|87274-128405 was predicted to encompass the *URA3* gene, but contain a substantial number of non-sequenced sites, we used another gene model scaffold311.1|size6309|122017-128325, which was predicted mainly by using transcriptome data (Supplementary Fig. S3A). We designed specific primers for amplifying the gene by PCR and sequencing (Table S1).

We extracted DNA from SSB01 and T01. Cells were collected by centrifugation twice at 5,000 × g for 5 min at 25 °C. Culture medium was discarded, and the cell pellet was re-suspended in 500 µl of MilliQ water (Merck Millipore, Germany). An equal volume of phenol/chloroform/isoamyl alcohol (25:24:1) (Nippon Gene, Japan) and approximately 20 µl of glass beads (Sigma-Aldrich, Merck Millipore, Germany) were added and mixed well by vortexing. After centrifugation, the clear upper aqueous phase containing DNA was carefully transferred to a new microtube and DNA was precipitated by adding an equal volume of isopropanol and 1/10 volume of 3 M sodium acetate. After centrifugation, the DNA pellet was washed with 70% ethanol, dried and dissolved in TE. DNA quality and concentration were checked using a NanoDrop (Thermo Fisher Scientific, Massachusetts, USA). The *URA3* gene fragments were obtained through nested PCR and by using four primers (1^st^, Ura3g_F1 and Ura3g_R1; 2^nd^, Ura3g_F2, and Ura3g_R2) using Tks Gflex DNA polymerase (Takara Bio, Japan). The cloned PCR products were sequenced using ABI 3130 DNA sequencer (Applied Biosystems, California, USA) and using 22 primers (Supplementary Table S1, except Ura3g_F1, Ura3g_R1, T7 and SP6m). The nucleotide sequences were deposited at DDBJ/EMBL/GenBank under accession numbers LC363939 and LC363940.

The *URA3* gene sequences were conceptually translated into proteins and used for multiple sequence alignment and phylogenetic analysis as described previously^28^. IQ-TREE was used to reconstruct phylogenetic trees using LG + F + G4 model^29^.

### Yeast mutant complementation

Synthetic cDNA sequences encoding *Symbiodinium* URA3 proteins, for which the codon usage was optimized for the budding yeast *Saccharomyces cerevisiae*, were designed using wild type and T01 mutant strains. The cDNA was digested with restriction enzymes *Bam*HI and *Pst*I (New England Biolabs, Massachusetts, USA) and cloned into the expression vector p414-TEF using T4 ligase (New England Biolabs, Massachusetts, USA) under the control of the *TEF1* (Translation Elongation Factor 1-alpha) promoter, which enables strong and constitutive expression. After amplified in *E. coli*, the vectors were purified and introduced into the yeast strain W303a (MATa ade2-1 his3-11, 15 leu2-3, 112 trp1-1 ura3-1 can1-100) by the lithium acetate method, followed by screening on plates lacking tryptophan. Resulting transformants possessing the empty vector, wild type *URA3* gene and mutant gene were cultured on plates containing or lacking uracil, and examined for complementation of the uracil requiring phenotype.

### Symbiosis experiments using the model sea anemone *Exaiptasia pallida*

*E. pallida* strain H2 was maintained at a density of 3 to 8 animals per plastic case (10 cm diameter), and filled with ASW. Aposymbiotic individuals were kept in dark and the symbiotic ones were maintained under a 12 h light: 12 h dark cycle, with lighting provided at an irradiance of approximately 20 µmol photons m^−2^ s^−1^, at 25 °C, and fed Clean white shrimp (Kyorin, Japan) 1 to 5 times per week.

Aposymbiotic *E. pallida* was transferred to a 6 well plate (1 individual/well) and maintained in the psnASWU medium, which was a mixture of psnASW (ASW containing 0.01 mg ml^−1^ of penicillin, 0.01 mg ml^−1^ of streptomycin, 0.02 mg ml^−1^ of neomycin) and 0.2 mg ml^−1^ of uracil, for a day prior to feeding. Aposymbiotic individuals were fed Clean white shrimp mixed with T01 (n = 6), or SSB01 (n = 3) as positive control, or no *Symbiodinium* cells (n = 3) as negative control. After feeding, *E. pallida* was pre-incubated in psnASWU for 7 days. After pre-incubation, psnAWSU was changed to psnASW. Three T01-fed individuals were kept in psnASWU for comparison. We observed individuals using fluorescence stereo microscopes (Leica, M205FA, Germany) and continued changing psnAWSU or psnAWS daily. Micrographs were processed and analysed using ImageJ software (NIH). For calculating symbiont area, images taken with a Green filter set (to visualize the chlorophyll autofluorescence of any *Symbiodinium* present) were processed by NIH ImageJ including LPixel ImageJ Plugins (LPX filter2d [filter = bandPassOps__, bpMode = Gaussian, low = 0, hi = 10, postProc = none], freely downloadable from https://lpixel.net/products/lpixel-imagej-plugins/) and threshold set by Otsu algorithm and area measurement was performed by Analyze Particles command (Size = 4-Infinity). Observation was discontinued when the symbiont area was saturated and each symbiont could not be distinguished. The rate of change in symbiont area was calculated as relative values in comparison to the area at the time of medium change from psnAWSU to psnASW. Student’s two-tailed t-tests were performed to test whether the area ratios significantly differed between the treatments.

## Electronic supplementary material

Dataset 1

Dataset 2
