# Supplementary material for: Isolation of uracil auxotroph mutants of coral symbiont alga for symbiosis studies
Source: Sci Rep. 2018 Feb 19;8:3237. doi: 10.1038/s41598-018-21499-3 (PMC5818653; doi:10.1038/s41598-018-21499-3)
Supplement: Supplementary file 1 — Dataset 1 [file 41598_2018_21499_MOESM1_ESM.zip › Supplementary_Table_Figs.Ishii_et_al.pdf]

Supplementary material to:

Isolation of uracil auxotroph mutants of coral symbiont alga for symbiosis studies

Yuu Ishii[1], Shinichiro Maruyama[1*], Konomi Fujimura-Kamada[2], Natsumaro Kutsuna[3,4], Shunichi Takahashi[2,5], Masakado Kawata[1*], Jun Minagawa[2,5]

1 Department of Environmental Life Sciences, Graduate School of Life Sciences, Tohoku University, Sendai, Miyagi, Japan

2 Division of Environmental Photobiology, National Institute for Basic Biology, Okazaki, Aichi, Japan

3 Department of Integrated Biosciences, Graduate School of Frontier Sciences, University of Tokyo, Kashiwanoha, Chiba, Japan

4 LPixel Inc., Bunkyo, Tokyo, Japan

5 Department of Basic Biology, School of Life Science, SOKENDAI (The Graduate University for Advanced Studies), Okazaki, Aichi, Japan

*** Corresponding authors**

E-mail: maruyama@tohoku.ac.jp, kawata@m.tohoku.ac.jp

Supplementary Table and Figures

Table S1. **Primers used for sequencing**

Primer name	Seq (5' to 3')	Start (scaffold311.1)
Ura3c_F1	TATGTGTGGGCCTTGATCCAC	122845
Ura3c_R1	ATCATTCTCATCTCGCCGCTC	127568
Ura3c_R2	TCCATGGCATCTACATCGGTTG	126328
Ura3g_F1	ATCACATCCCACCTCATGAACC	121308
Ura3g_F2	CATCACTGCAAAACTGATAAGGGCC	121968
Ura3g_F3	GAAGGGTTTTGGTGAAGGTTGAGGG	122434
Ura3g_F4	TTTTGGGTGCATCATAACGTCATGG	123246
Ura3g_F5	TGAACTTTACCAGCGCCCATTTT	123413
Ura3g_F6	TGCGAGTCTCTTCATTTTGGCAA	124468
Ura3g_F7	CATGGCTGTATGATATGAGCAACGC	124790
Ura3g_F8	TTTCATGGTGACTGGGCTGATAAGA	126683
Ura3g_F9	TTGGTTGATTCTCCCCACCA	126600
Ura3g_R1	CCCAGTTGTGTCATGCTCATTC	127724
Ura3g_R2	TGCTCATTCATTTACACCTGTCCTGA	127711
Ura3g_R3	ACCAAGACTAACAGTACGCACCT	126002
Ura3g_R4	CTTTAATGACAATGGCCCGGCTA	125045
Ura3g_R5	CCACCTCCACAGAAAGAACATGC	124041
Ura3g_R6	AAGTTCGTTGTAAAGCTTCCCAACC	123018
Ura3g_R7	TGGATTCAATCATTTCACCAACCCA	125583
Ura3g_R8	TCCAACCTCTAGATATTGGCAGCAA	127141
Ura3g_R9	GGTTAATAAGCCCGGCCTTG	121729
Ura3g_R10	CTAGTGAGGAGTTTTGGCGC	122024
Ura3g_R11	TCAAACAAGACATGCCAGCC	122141
Ura3g_R12	TCCTCAATGAGATGCTGGCA	122282
T7	TAATACGACTCACTATAGGG	
SP6m	CTCCCATATGGTCGACCTGC	

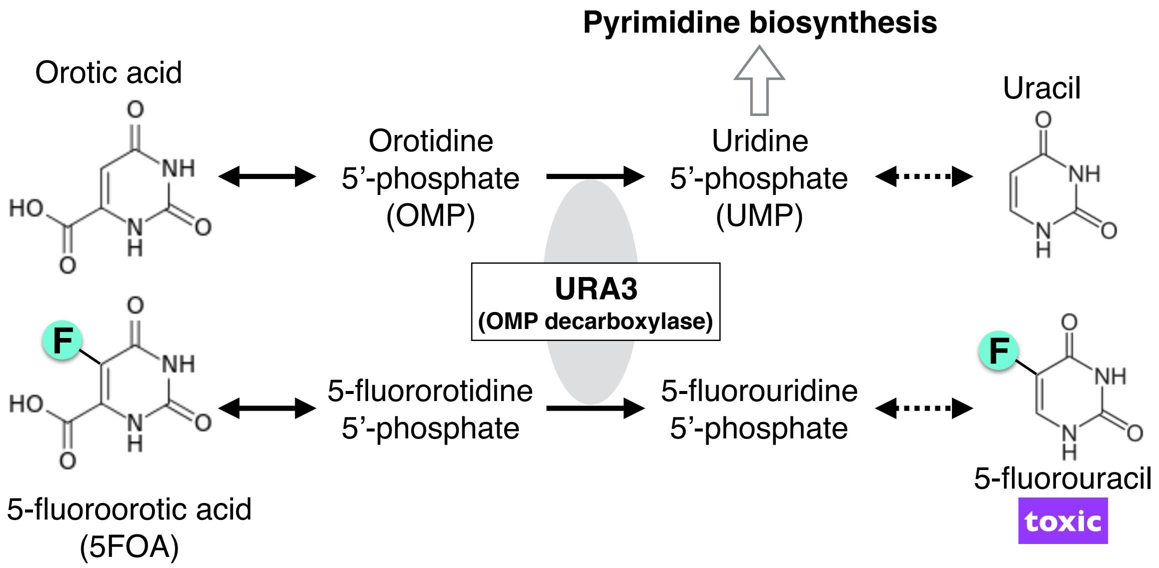

Supplementary Fig. S1**. Reactions catalysed by OMP decarboxylase, the *URA3* gene product.** Cells possessing active *URA3* that encodes OMP decarboxylase can convert OMP to UMP, and are sensitive to 5FOA due to the lethal toxicity of 5-fuorouracil, while mutants possessing non-functional *URA3* gene are unable to synthesize pyrimidine derivatives, but are resistant to 5FOA.

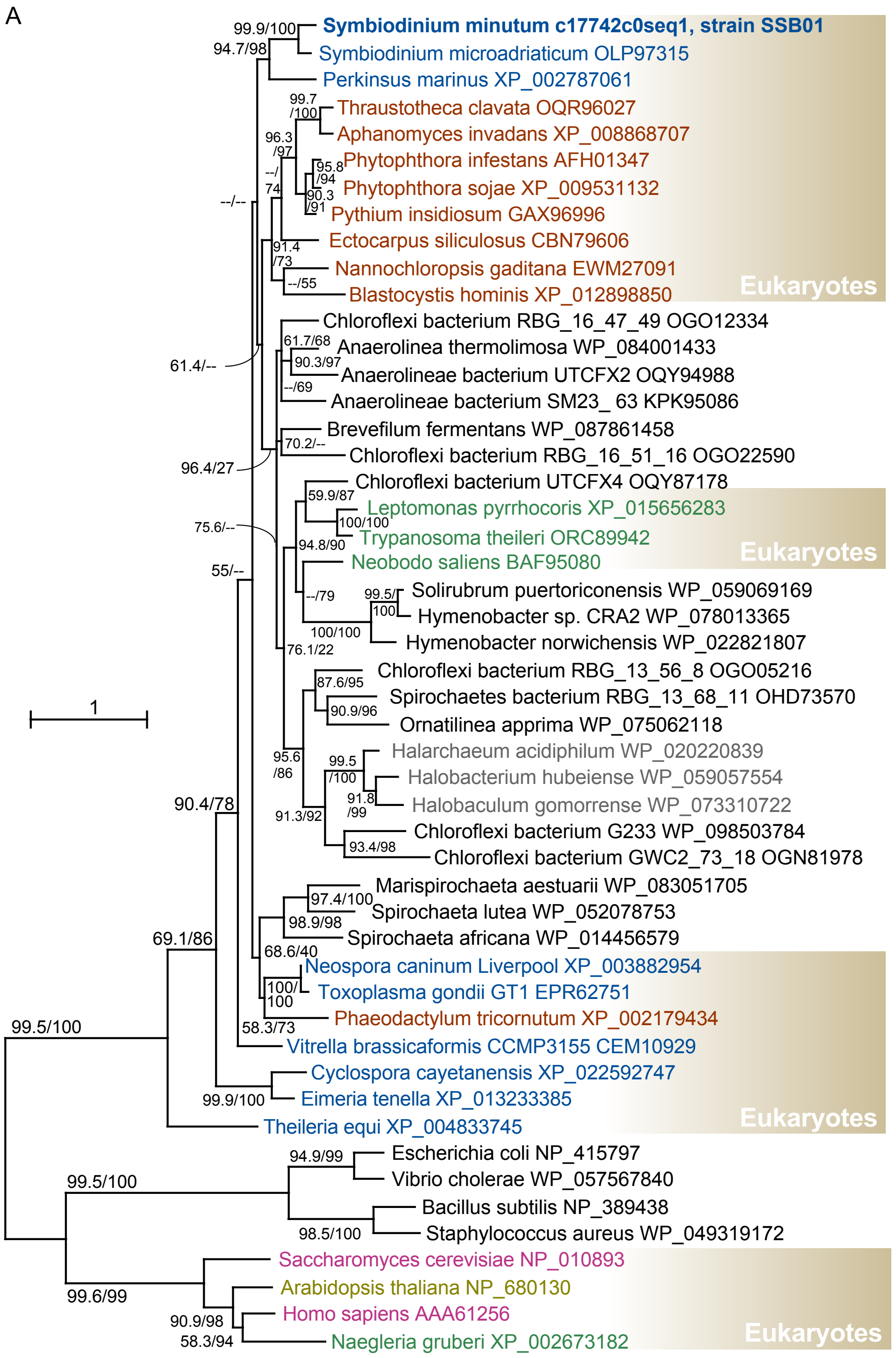

Supplementary Fig. S2. **Sequence analysis of URA3 proteins**

A. Phylogenetic tree of URA3 proteins. Support values using SH-like approximate likelihood ratio test (left) and ultrafast bootstrap approximation (right) are shown on each branch. Text colors represent phylogenetic domains or eukaryotic super-groups to which the organisms belong. Black, Bacteria; gray, Archaea; blue, Alveolata; brown, Stramenopiles; green, Excavata; olive, Archaeplastida; magenta, Opisthokonta. A single copy *URA3* homolog was found in *Symbiodinium kawagutii* (Skav218766) but not used due to its highly divergent and non-alignable sequence.

B

```
                    1
Sy.T01              MSFFEKLTKR AKEIDSLLCV GLDPHKSELQ EDSAEGAFRF CQHLIE---D VACAFKPNAA
Sy.SSB01            MSFFEKLTKR AKEIDSLLCV GLDPHKSELQ EDSAEGAFRF CQHLIEETKD VACAFKPNAA
Sy.micro.      (294)MSFFEKLTKR ARDADSLLCV GLDPHKAELQ EDSAEGAFRF CSRLIEETKD LVAAYKPNAA
B.subtilis          ---------- --MKNNLPII ALDFA----- --SAEETLAF LAPFQQ---- EPLFVKVGME
E.coli              MTLTASSSSR AV-TNSPVVV ALDYH----- --NRDDALAF VDKIDP---- RDCRLKVGKE
H.sapiens      (225)HPVASKLLRL MQKKETNLCL SADVS----- --LARELLQL ADALGP---- SICMLKTHVD
Sa.cerevisiae   (15)SPVAAKLFNI MHEKQTNLCA SLDVR----- --TTKELLEL VEALGP---- KICLLKTHVD

                   61
Sy.T01              FFEAYGSAGW EALQRTLQLI P-KEIPIVLD AKRGDIGSTS E-AYATSAFS TLACDSITAS
Sy.SSB01            FFEAYGSAGW EALQRTLQLI P-KEIPIVLD AKRGDIGSTS E-AYATSAFS TLACDSITAS
Sy.micro.           FFEAYGVEGW GALQRTLALI P-KDIPIVFD AKRGDIGSTS E-AYACSAYQ TLNCDSVTVS
B.subtilis          LFYQEG---- --PSIVKQLK E-RNCELFLD LKLHDIPTTV N-KAMKRLAS LGV--DLVNV
E.coli              MFTLFG---- --PQFVRELQ Q-RGFDIFLD LKFHDIPNTA A-HAVAAAAD LGVW--MVNV
H.sapiens           ILNDFT---L DVMKELITLA KCHEFLIFED RKFADIGNTV KKQYEGGIFK IASWADLVNA
Sa.cerevisiae       ILTDFSMEGT VKPLKALS-A K-YNFLLFED RKFADIGNTV KLQYSAGVYR IAEWADITNA

                  121
Sy.T01              PYLGGDGLQP FLKDAS---- ---RGVWVLC KTSNPGSQDI QALELPTGEP LYLHVAKLCF
Sy.SSB01            PYLGGDGLQP FLKDAS---- ---RGVWVLC KTSNPGSQDI QALELPTGEP LYLHVAKLCF
Sy.micro.           PYLGSDGLQP FLKDPS---- ---RGAWVLC KTSNPGSQDI QALQLPSGEP LYVHVAKVCC
B.subtilis          HAAGGKKMMQ AALEGLEEGT PAGKKRPSLI AVTQLTSTSE QIMKDELLIE KSLIDTVVHY
E.coli              HASGGARMMT AAREALVPFG ---KDAPLLI AVTVLTSMEA SDL-VDLGMT LSPADYAERL
H.sapiens           HVVPGSGVVK GLQEVGLPLH ---RGCLLIA EMSSTGSQAT G--------- -----DYTRAA
Sa.cerevisiae       HGVVGPGIVS GLKQAAEEVT KEPRGLLMLA ELSCKGSLAT G--------- ----EYTKGT

                  181
Sy.T01              GTWAKEHQNA GLVVGATDVD AMEKIRAALP DVWFLSPGIG AQGGDLAKAL KVGLREDGLG
Sy.SSB01            GTWAKEHQNA GLVVGATDVD AMEKIRAALP DVWFLSPGIG AQGGDLAKAL KVGLREDGLG
Sy.micro.           LQWAKEHNNA GLVVGATDVE AMRAIRAAVP DVWFLSPGIG AQGGDLEAAL TAGLRPDGLG
B.subtilis          SKQAEESGLD GVVCSVHEAK AI--YQAVSP SFLTVTPGIR MSEDAANDQV RVATPAIARE
E.coli              AALTQKCGLD GVVCSAQEAV RF--KQVFGQ EFKLVTPGIR PQGSEAGDQR RIMTPEQALS
H.sapiens           VRMAEEHSE- -FVVGFISGS RV----SMKP EFLHLTPGVQ LEAGGDNLGQ QYNSPQEVIG
Sa.cerevisiae       VDIAKSDKD- -FVIGFIAQR DM-GGRDEGY DWLIMTPGVG LDDKGDALGQ QYRTVDDVVS

                  241
Sy.T01              I----LLPIS RGI-SKAENP KKMAETFR-A EINAL----R AAR
Sy.SSB01            I----LLPIS RGI-SKAENP KKMAETFR-A EINAL----R AAR
Sy.micro.           I----LLPIS RGI-SKAESP RKAAEAFR-D EINALRAKRT ASS
B.subtilis          K-GSSAIVVG RSI-TKAEDP VKAYKAVR-L EWE------G IKS
E.coli              A-GVDYMVIG RPV-TQSVDP AQTLKAIN-A SLQ------R SA-
H.sapiens           KRGSDIIIVG RGI-ISAADR LEAAEMYRKA AWEAYLSRLG V--
Sa.cerevisiae       T-GSDIIIVG RGLFAKGRDA KVEGERYRKA GWEAYLRRCG QQN
```

Supplementary Fig. S2. **Sequence analysis of URA3 proteins** (continued)

B. Multiple amino acid sequence alignment of URA3 proteins. Star and triangle indicate a conserved lysine residue and the deletion site found in T01, respectively. Proteins from *Symbiodinium* sp. T01 (sequenced in this study), *Symbiodinium* sp. SSB01 (identical to *Sy. minutum* contig comp17742_c0_seq1), *Sy. microadriaticum* (accession number: OLP97315), *Bacillus subtilis* (NP_389438), *Escherichia coli* (NP_415797), *Homo sapiens* (AAA61256), *Saccharomyces cerevisiae* (NP_010893) were used to construct the alignment.

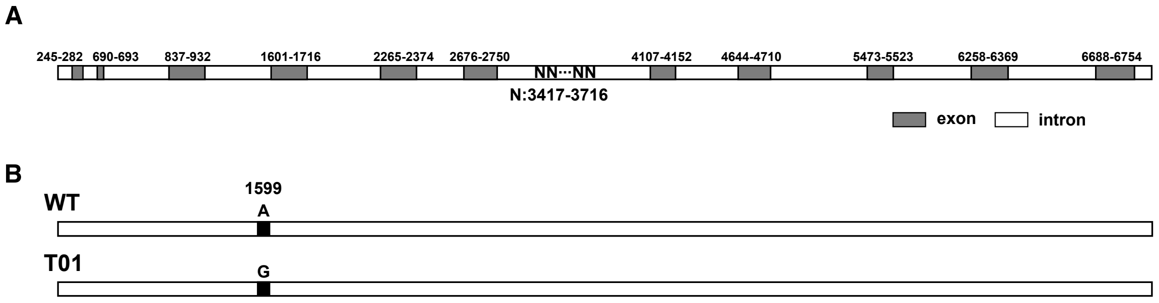

Supplementary Fig. S3. **Genomic sequences of *URA3* gene in wild type and T01**

A. WT *URA3* gene structure based on the gene model of *S. minutum* Mf1.05b is shown.

B. In T01, the 1599th base has been substituted from A to G.

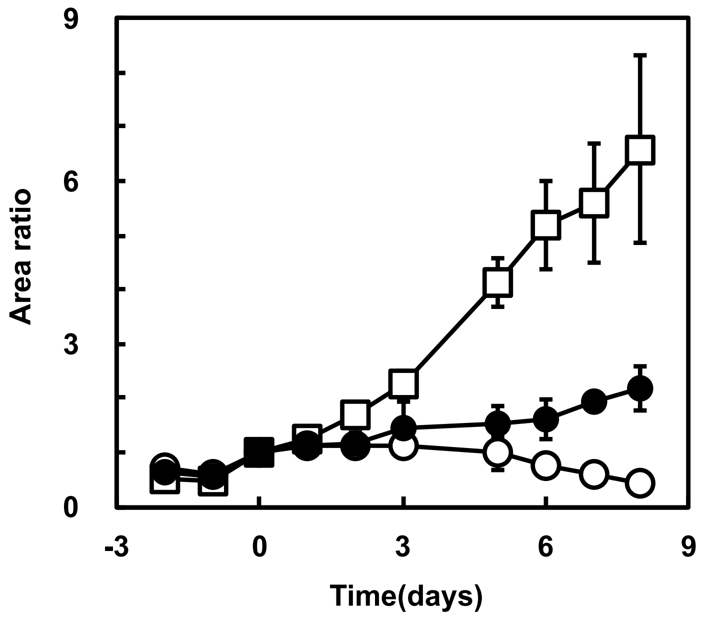

Supplementary Fig. S4. **Effect of uracil on the symbiotic states of wild type and T01**

To examine the effect of uracil on the symbiotic states of WT and T01, *Symbiodinium* area ratios were quantified by comparing signal regions of chlorophyll autofluorescence and normalized to the value of day 0 in the presence (T01, closed circles) or absence (T01, open circles; WT, open square) of uracil.
